# Shining a Light on Dark Sequencing: Characterising Errors in Ion Torrent PGM Data

**DOI:** 10.1371/journal.pcbi.1003031

**Published:** 2013-04-11

**Authors:** Lauren M. Bragg, Glenn Stone, Margaret K. Butler, Philip Hugenholtz, Gene W. Tyson

**Affiliations:** 1 Australian Centre for Ecogenomics, School of Chemistry and Molecular Biosciences, The University of Queensland, St Lucia, Queensland, Australia; 2 Advanced Water Management Centre, The University of Queensland, St Lucia, Queensland, Australia; 3 CSIRO Mathematics, Informatics and Statistics, Queensland, St Lucia, Australia; 4 School of Computing and Mathematics, University of Western Sydney, Parramatta, New South Wales, Australia; ETH Zurich, Switzerland

## Abstract

The Ion Torrent Personal Genome Machine (PGM) is a new sequencing platform that substantially differs from other sequencing technologies by measuring pH rather than light to detect polymerisation events. Using re-sequencing datasets, we comprehensively characterise the biases and errors introduced by the PGM at both the base and flow level, across a combination of factors, including chip density, sequencing kit, template species and machine. We found two distinct insertion/deletion (indel) error types that accounted for the majority of errors introduced by the PGM. The main error source was inaccurate flow-calls, which introduced indels at a raw rate of 2.84% (1.38% after quality clipping) using the OneTouch 200 bp kit. Inaccurate flow-calls typically resulted in over-called short-homopolymers and under-called long-homopolymers. Flow-call accuracy decreased with consecutive flow cycles, but we also found significant periodic fluctuations in the flow error-rate, corresponding to specific positions within the flow-cycle pattern. Another less common PGM error, high frequency indel (HFI) errors, are indels that occur at very high frequency in the reads relative to a given base position in the reference genome, but in the majority of instances were not replicated consistently across separate runs. HFI errors occur approximately once every thousand bases in the reference, and correspond to 0.06% of bases in reads. Currently, the PGM does not achieve the accuracy of competing light-based technologies. However, flow-call inaccuracy is systematic and the statistical models of flow-values developed here will enable PGM-specific bioinformatics approaches to be developed, which will account for these errors. HFI errors may prove more challenging to address, especially for polymorphism and amplicon applications, but may be overcome by sequencing the same DNA template across multiple chips.

## Author Summary

DNA sequencing is used routinely within biology to reveal the genetic information of living organisms. In recent years, technological advances have led to the availability of high-throughput, low-cost DNA sequencing machines (‘sequencers’). In 2011, Life Sciences released a new sequencer, the Ion Torrent Personal Genome Machine (PGM). This is the first sequencer to measure changes in pH rather that emitted light to register sequencing reactions. Consequently, this unique technology is both cost-effective and advertised to have high accuracy, making it attractive for many laboratories. However, every sequencing technology introduces unique errors and biases into the resulting DNA sequences, and understanding PGM-specific characteristics is crucial to determining suitable applications for this new technology. We comprehensively examine the types of errors and biases in PGM-sequenced data across several experimental variables, including chip density, template kit, template DNA and across two machines. Using statistical approaches, we quantify the influence of experimental variables, as well as DNA sequence-specific effects, and find that the PGM has two types of technology-specific errors. We also find that the accuracy of the PGM is poorer than that of light-based technologies, and we make recommendations for this technology as well as provide statistical models for overcoming PGM sequencing errors.

## Introduction

The last decade has seen dramatic advances in sequencing technology that have relied on highly-parallel optical sensing of polymerisation reactions. These advances have substantially reduced sequencing costs, however further reduction in cost is limited by the dependence of these platforms on photo-receptive sensors and their associated reagents. In 2011, Life technologies began distribution of the Ion Torrent Personal Genome Machine (PGM). The PGM leverages advances in semi-conductor technology and ion-sensitive transistors to sequence DNA using only DNA polymerase and natural nucleotides, with each polymerisation event recognised by pH changes alone [1].

The PGM requires similar library preparation steps to Roche 454 shotgun libraries, where an adapter and key () is ligated to the DNA templates, and under optimal conditions, a single DNA template is affixed to a bead and clonally amplified using emulsion PCR. The beads are then loaded onto the chip, where, on average, each well contains less than a single bead.

Deoxynucleotide triphosphates (dNTPs) are flowed over the surface of the bead in a predetermined sequence, with zero or more dNTPs ligating during each flow. While the first generation of the PGM cycled through the four nucleotides in a step-wise fashion (as does the Roche 454 pyrosequencer), this cycle was modified to have a period of 32, with a pattern that repeats some nucleotides in a period shorter than four. This more complex flow cycle, referred to as the *Samba*, was implemented to improve synchronicity of clonal templates on the bead at the cost of a flow-sequence not optimised for read length. A single proton is released for every nucleotide incorporated during the flow, resulting in a net decrease of the pH in the surrounding solution. This pH change is measured by an ionic sensor and then converted to a flow value using a physical model of the cell and its contents. The PGM base-caller takes these flow-values and corrects for phase and signal loss, and also normalizes the raw flow-values to the key sequence (the key is a known four-base DNA molecule appended to the 5′ region of every read sequence). Finally, quality assurance procedures are applied to the data, filtering polyclonal and noisy reads, and clipping adapters and low quality regions from the 3′ end of the remaining reads.

An assessment of the PGM sequencing technology was provided with the publication of the sequencing platform [1]. However, this analysis was conducted using quality-trimmed data (rather than raw reads) and reported accuracy based on a consensus rather than individual read accuracy. In addition, substantial changes have been made to the platform since the initial publication, including the change of flow order (*Samba*), quality control and base calling, which are likely to influence the error rates and biases in PGM data. Two studies comparing benchtop sequencers, including the PGM, were recently published [2], [3], both focusing on the assembly prospects of benchtop platforms on single isolate genomes. Consistent with the scope of these studies, they used the quality-trimming recommended by each distributor and did not generate sufficient replicate data to understand variability in accuracy. No study of PGM data to date has attempted to robustly evaluate error rates or explored the possible causal factors of errors at the flow-level. Here, we characterise the nature of errors produced by the PGM, including sequencing kit, chip type, machine variability, template G+C%, and chip, read and flow positional effects. Our findings contribute to a more comprehensive understanding of the errors and biases inherent in PGM data, and will enable the bioinformatics community to develop appropriate algorithms for this new platform.

## Results

### Datasets

In this study, we considered the influence of both technological (template preparation kit, chip, machine) and DNA template factors on sequencing output using a full factor statistical design (**see** **Methods**). We evaluated three template preparation kits, listed in chronological release order, Ion OneTouch Template Kit (100 bp OneTouch kit), Ion Xpress Template 200 Kit (200 bp manual kit), Ion OneTouch 200 Template Kit (200 bp OneTouch kit), two chip densities, 314 (1.2M sensors, density 200,000 reads) and 316 (6.1M sensors, density 1 million reads) and two PGM machines (named here *a* and *b*). Two species, *Sulfolobus tokodaii* (33 G+C%) and *Bacillus amyloliquefaciens* (46% G+C%), both with relatively small, sequenced genomes and no extra-chromosomal DNA, were selected to represent different G+C% templates. A third organism, *Deinococcus maricopensis* (69% G+C), was selected for its high G+C content, however library preparation consistently failed using the 200 bp kits, and read throughput was extremely low for the 100 bp OneTouch kit, consequently it was excluded from subsequent analyses. Fifteen datasets were generated (**Table 1**) and used to evaluate the effects and interactions between template preparation kit, chip and machine on the measured error rate (**see** **Methods**).

**Table 1:** Sequencing runs generated for this study.

Treatment	# Reads	% Wells with ISPs	% With adaptors	#Reads Used	% Reads mapping	Mean length	Mean Length (AT)	Mean quality
314-B-a-100	275,058	54%	96%	275,058	96%	146.8	116.1	32.9
314-S-a-100	188,925	63%	94%	188,925	94%	153.6	125.2	32.5
316-S-a-100	2,135,728	55%	97%	300,000	97%	149.9	121.4	33.5
316-B-a-100	2,364,054	67%	98%	300,000	98%	145.2	114.2	33.1
314-B-a-200M	453,539	66%	100%	300,000	95%	267.7	248	33.6
314-S-a-200M	286,106	45%	100%	286,106	94%	269.40	254.5	33.9
316-S-a-200M	1,321,709	51%	100%	300,000	91%	268.40	254.2	30.9
316-S-b-100	1,442,952	51%	98%	300,000	98%	151.3	121.4	32.7
314-B-b-100	441,037	68%	92%	300,000	91%	152.9	123.3	33.7
316-B-a-200M	1,124,128	95%	100%	300,000	92%	267.4	248.2	29.5
314-S-b-200M	197,811	36%	100%	197,811	93%	271.7	256.7	33.4
316-B-b-200	2,345,739	95%	5.7%	300,000	94%	298.9	250.5	33.2
314-S-b-200	426,411	94%	15%	300,000	85%	318.2	278.4	34.0
316-S-a-200	2,586,746	82%	6.7%	300,000	93%	305.0	258.5	32.1
314-B-a-200	373,256	50%	12%	300,000	87%	307.9	263.9	34

The name for each run is comprised of the chip (314, 316), species (B – *Bacillus amyloliquefaciens*, S – *Sulfolobus tokodaii*), machine (a, b), and kit (100 - Ion OneTouch Template Kit, 200M - Ion Xpress Template 200 kit, 200 - Ion OneTouch 200 Template kit). Runs are listed in chronological order. ‘% Wells with ISPs’ describes the percentage of wells on the chip which contained a bead. Mean Length AT denotes length after 3′ adapter trimming.

For the 100 bp OneTouch kit and 200 bp manual kit, the great majority of reads (94–100%) had adapter sequences detected by the PGM software. The newest kit considered here, the 200 bp OneTouch Kit, had a very low percentage of reads with detected adapters (7–12%), which may be due to library construction differences (longer inserts used). We applied the 3′ clipping of adapter sequences as specified in the Standard Flowgram File (SFF), however we did not apply the recommended 3′ quality clipping – allowing more realistic calculation of raw error rates. Datasets larger than 300,000 reads were randomly subsampled down to 300,000 reads as this provided sufficient information for downstream analyses (**Table 1**).

On average, 93±3.5% of all analysed reads mapped to their respective reference genomes, with the poorest performance obtained with the 200 bp OneTouch kits on 314 chips. Mean read quality across all the datasets was 32.88±1.20 s.d.

The distribution of read lengths (**Figure S1**) shows that both the 100 bp and 200 bp One Touch kits were bi-modal with a smaller secondary peak ∼100 bp greater than the expected length, which was more prominent in runs using the 314 chip. These longer reads did not exhibit any deviation in mean G+C content or homopolymer composition from the dominant read length peak for their respective runs. However the majority of these longer reads did not map to the reference genome, but those that could be mapped had an error rate double that of the mean base-error rate across all datasets. Considering these reads have a substantially higher error rate, we recommend the removal of unexpectedly long reads prior to analysis.

### High frequency polymorphisms between sequenced reads and reference genomes

Prior to assessing PGM error profiles, we determined if there were any genuine polymorphisms between the PGM determined and reference genomes, which may be the result of accumulated mutations in the genome [4] or sequencing errors in the original genome project. We tested each base difference between the reads and their respective reference genome to identify whether the number of observed differences was significantly higher than the expected error rate (**see** **Methods**). Across all datasets, there were a large number of significant differences, predominantly high-frequency insertion and deletion (indel) polymorphisms (**Table 2**). While the number of polymorphisms appear to be lower for 100 bp OneTouch kits, this is likely due to lower coverage reducing the sensitivity of our ‘polymorphism’ detection. It has been previously reported that the majority of indel polymorphisms detected in PGM reads are false-positives, even when the ‘putative’ indel was present across a large number of reads [3], [5]. We would expect that if the indels in our datasets were *bona fide* polymorphisms they would be observed across all datasets for the same species. Analysis of the 200 bp kits revealed that 87% of high-frequency indels across the *B. amyloliquefaciens* datasets, and 82% across *S. tokodaii* were unique to a single run (**Figure S2a and S2b**). As the data were derived from the same DNA template, this strongly indicates that these indels are due to PGM-based error as opposed to genuine polymorphisms. While few high frequency indel (HFI) sites were shared amongst all 200 bp runs for the same species, the size of the intersection between pairs of runs suggests that the HFI (or a subset of them) are not random (i.e. they may be more prevalent in or around a particular sequence motif) (**Figure S2a and S2b**). HFIs have been observed previously in PGM data, with some evidence to suggest the HFI were asymmetrically distributed across reads in the forward versus reverse orientation (or vice versa) [3]. We investigated whether any of the HFIs in our data were asymmetrically distributed across the forward and reverse oriented reads aligning across that site (**see** **Methods**). This test was only performed on the 200 bp kits as the 100 bp kits had insufficient coverage for evaluation. Significant indel-strand asymmetry was detected in 7.4% (897/12,107) of the testable putative indel sites across the 200 bp datasets (**Table 2**), with some datasets having a substantially higher percentage of asymmetric indels than others (i.e. 314-S-b-200, 314-S-a-200M). As with the HFI superset, these strand-asymmetric HFIs have more sites in common between runs than expected for a random event (**Figure S2c and S2d**).

**Table 2:** Number of genomic locations where a significant proportion of reads disagreed with the reference.

*Treatment*	*Substitutions*	*Deletion*	*Insertion*	*Asymmetric Deletion (# significant/# tested)*	*Asymmetric Insertion (#significant/#tested)*
314-B-a-100	24	107	40	N/A	N/A
314-S-a-100	18	45	38	N/A	N/A
316-S-a-100	21	162	98	N/A	N/A
316-B-a-100	9	110	79	N/A	N/A
314-B-a-200M	553	761	1303	12/618	5/644
314-S-a-200M	483	1105	2446	165/365	33/529
316-S-a-200M	568	337	911	35/247	30/561
316-S-b-100	13	131	161	N/A	N/A
314-B-b-100	11	92	83	N/A	N/A
316-B-a-200M	534	246	546	1/159	0/294
314-S-b-200M	309	409	1014	24/365	13/529
316-B-b-200	164	979	717	32/905	0/315
314-S-b-200	292	1579	2189	398/1533	21/1041
316-S-a-200	198	398	1100	64/350	7/623
314-B-a-200	170	1127	1214	53/1049	4/554

The last two columns show the number of significant strand-specific error instances out of the total testable instances (testable defined here as having reads aligning in both orientations over the site).

The majority of HFI errors were single-base insertions or deletions and only 29% of these occurred in homopolymeric regions, in contrast, 82% of strand-asymmetric HFIs occurred in a homopolymer of length two to three. In general, HFI errors manifested most commonly as insertions of A/T or deletions of C/G (**Figure S3**a). Strand-asymmetric HFIs were dominated by deletions of C and G (**Figure S3b**). There was no relationship between the base or flow positions of these errors across the reads with the same HFI. It will require further experimentation to identify whether HFIs are introduced by the library preparation, template preparation or the sequencer itself. The HFI error rate relative to the reference genome was approximately 1 in 1000 to 1 in 2000 bp, and HFIs accounted for 0.03%–0.09% of all bases in each dataset. Given that it is difficult to distinguish HFIs from *bona fide* polymorphisms without sequencing the same template over multiple chips, PGM sequencing may be compromised in polymorphism detection and amplicon sequencing projects. Here, we mask the HFIs from downstream analyses, as they are unlikely to be the consequence of individual flow-call inaccuracy, and will introduce bias into modeling of base and flow-call accuracy.

### Coverage and G+C bias

**Figure 1:**
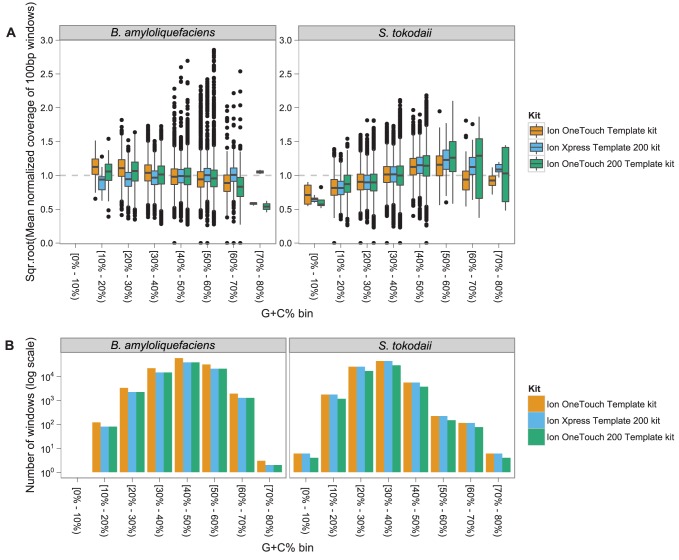
Relationship between G+C% and the observed mean coverage for 100 bp bins in the reference genome. Panel (a) is a boxplot of the distribution of the square-root normalized mean read depth across the 100 bp windows for each reference genome, broken down further by sequencing kit and G+C% bin. The coverage for each run was normalised by the mean coverage –the boxplots show the square-root fold-change from the mean genomic coverage for each combination of species, kit and G+C% bin. Thus a value of 2 means the coverage was four times that of the mean for that sequencing run. The boxes display the central 50% of the values in each treatment, with the median represented by the solid black horizontal bar. The whiskers each extend for 1.5× the inter-quartile range, and the black dots represent extreme individual observations which fall outside this range. The variability observed in the high G+C bins are likely due to the small sample size for these G+C regions, shown in panel (b). The outliers are potentially due to repetitive content in the genome that failed to be masked by our perfect match repeat approach.

Initial studies characterising the PGM reported that there was little correlation between genomic coverage and G+C% content. More recently it was claimed that, based on visual comparisons of the theoretical versus empirical genomic coverage, there was substantially less read coverage in A+T rich regions [3]. Here, we attempt to quantify the magnitude and significance of the relationship between coverage and G+C content. Inspection of the data suggested that the relationship between G+C% and coverage differs for the two species used in this study (**Figure 1a**). While *B. amyloliquefaciens* has a higher mean G+C (46.1%) compared to S. *tokodaii* (32.8%), both have a large number of 100 bp windows within the range of 20–50% G+C content, allowing direct comparison (**Figure 1b**). The positive correlation between G+C and coverage in *S. tokodaii* versus the negative correlation with *B. amyloliquefaciens* is clear even when restricting to this 20–50% G+C range. This suggests that the relationship between G+C% and coverage is influenced by the DNA template from which the sequences are derived. We fitted a linear model to the normalised coverage to evaluate the significance and magnitude of the relationship between G+C% and coverage, as well as the influence of species (DNA template) on this relationship (**see** **Methods**). All terms in the regression were significant (p<0.0001), and inspection of the linear model diagnostic plots revealed no dramatic deviations from normal model assumptions (**Figure S5**). This model can be split into two linear regressions, one for *B. amyloliquefaciens*,
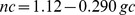
and the second for *S. tokodaii*,
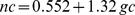
where 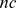 is the *normalised* coverage and 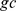, the proportion G+C% in a 100 bp window. The *B. amyloliquefaciens* model describes a small negative effect for increasing G+C% on coverage, whereas *S. tokodaii* has a larger, positive effect. This relationship requires further investigation with a wider range of species, but it is replicated across the various kits, chips and machines used in this study. While we had originally intended to include the high G+C% organism, *Deinococcus maricopensis* (**Methods**), the inconsistency in read throughput across chips for this species necessitated its exclusion. However, we analysed the highest throughput run for *D. maricopensis* (kit - 200 bp Manual Kit, chip - 316) and observed an extreme bias against high G+C% regions, with lower G+C content regions (45%) having 2.5× the mean genomic coverage, but higher G+C regions (80%) receiving only 0.2× the mean coverage (**Figure S6**).

Both PCR and gel extraction processes have been implicated in G+C% coverage biases in Illumina sequencing [6], [7]. PCR has been shown to preferentially amplify mid-G+C% fragments, and size selection using gel extraction approaches, which heat the DNA, often leading to underrepresentation of low G+C% sequences. In this study, size selection was performed using the Pippin Prep (**see** **Methods**) circumventing gel extraction, leaving the PCR steps as the likely cause of the coverage biases observed in low and high G+C% regions. As PCR biases are exerted only on sequences processed together, it is expected that the observed relationship between G+C% and coverage will depend on the mean and range of G+C% in the DNA template. As demonstrated here, organisms with 100 bp G+C% windows within the 20%–80% G+C range will experience little bias in coverage, whereas organisms with G+C% regions outside this ‘safe’ range will suffer substantial G+C biases. The G+C% bias appears asymmetric, with the bias against high G+C% regions (i.e. *D. maricopensis*) more pronounced than low G+C% regions (i.e. *S. tokodaii*). In fact this bias against extremely high G+C% templates may explain why sequencing *D. maricopensis* libraries, as well as *Streptomyces sp.* and *Burkholderia* sp. libraries, either failed during library preparation or produced insufficient sequence data. PGM sequencing of organisms with low or high G+C% will experience substantial read-depth biases, however, more concerning is this failure to produce libraries from high G+C% templates. This bias will likely result in inaccurate representation of organisms in metagenomic and metatranscriptomic data generated on the PGM.

### Replicate bias

The artificial introduction of large groups of exact or near exact duplicate reads which share the same start site was first observed in Roche 454 pyrosequencing data [8]. This ‘replicate bias’ is generally attributed to issues within the emulsion PCR (emPCR) step [9], [10]. Ion Torrent library preparation also uses emPCR, making it potentially susceptible to this bias. For each run, we evaluated the distribution of read start positions, and found no obvious deviations from the uniform distribution. Consistent with Rothberg et al. [1], we find that despite the use of emulsion PCR, PGM sequences do not exhibit replicate bias.

### Overview of PGM sequencing error

**Figure 2:**
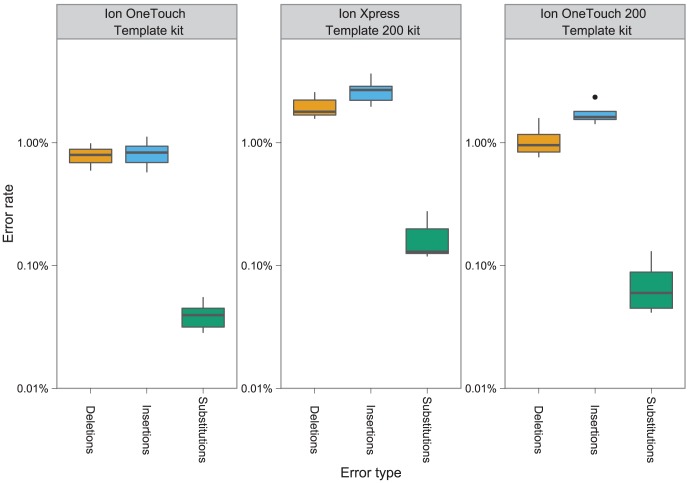
Mean rates of insertion, deletion and substitution errors across the three sequencing kits. Each box-plot shows the distribution of error rates for the specified type across the runs for the specified kit (species are aggregated).

Across the three kits, insertion/deletion (indel) errors were the dominant form of error in the reads (**Figure 2**). Insertions were more common (0.84%, 2.69%, 1.76% of untrimmed bases in the 100 bp One Touch, 200 bp Manual, and 200 bp One Touch) than deletions (0.80%, 1.98%, 1.07% of untrimmed bases in the 100 bp One Touch, 200 bp Manual, and 200 bp One Touch). Substitutions errors were an order of magnitude less frequent than insertion/deletion errors, with a mean rate of 0.04%, 0.17% and 0.07% in the 100 bp OneTouch, 200 bp Manual and 200 bp OneTouch kits. In total, the mean error rate for each kit based on the sum of these error types is 1.68%, 4.84% and 2.90% for the 100 bp OneTouch, 200 bp manual and 200 bp OneTouch kits, with homopolymer errors responsible for between 96–97% of the total error. Substitution errors had the highest variation in frequency, with a standard deviation ranging from 26%–56% of the mean substitution rate. The standard deviation of indel errors was between 19%–34% of the mean indel rates. The raw indel error rates observed in our study (1.62%, 4.66% and 2.83% for each of the 100 bp One Touch, 200 bp Manual and 200 bp OneTouch kits) are substantially higher than reported for quality trimmed data (100 bp OneTouch – 1.1% [1] using simple flow-cycle, 1.5% [2] using Samba, 1.78% [3] for the 200 bp manual using Samba flow-cycle). We attribute these differences to two factors, (1) our indel tolerance in read mapping which allows more of the data to map to the reference, and (2) the effect of quality trimming (discussed below).

**Figure 3:**
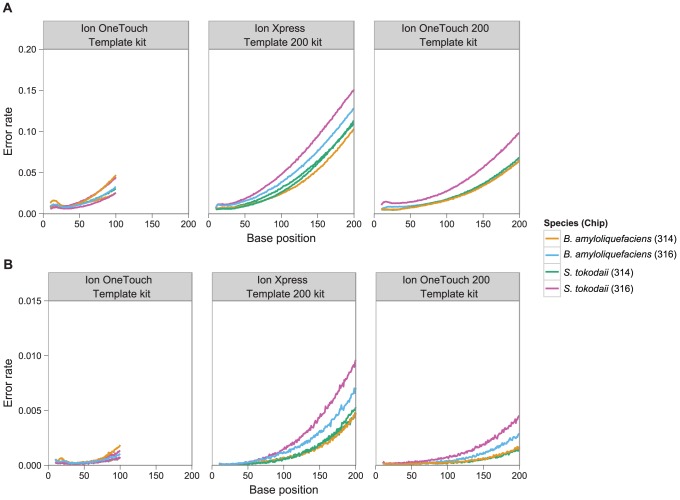
Relationship between base position and error rate for homopolymer (over-call/under-call) versus substitution errors. Panel (a) shows the homopolymer error rate (insertion+deletion) by read base position, and panel (b) shows the substitution error rate by base position. Each line is the raw mean error rate for a single data-set with the kit and species as specified by the colour key.

Similar to Roche 454 pyrosequencing [11], [12], indel-error rates increase with distance from the read start (**Figure 3a**). We also find that the substitution error rate increases towards the 3′ end of the read (**Figure 3b**). Error rates were higher on the 316 chips, and the low G+C% organism (*S. tokodaii*) had a higher error rate than the mid G+C% organism (*B. amyloliquefaciens*) across all datasets. The high mean indel error rate in the 200 bp manual kit appears to be due to a more rapid deterioration in base calling accuracy along the length of the read relative to the 200 bp OneTouch kit (**Figure 3a**).

### Homopolymer errors

**Figure 4:**
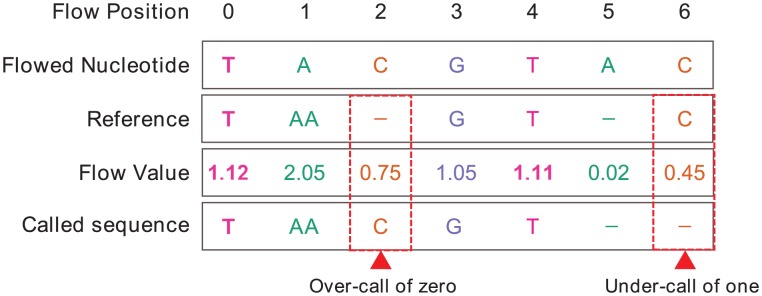
Examples of over-call/under-call errors in homopolymers of length less than 2. By aligning the read (derived from the rounded flow-values), and its corresponding reference sequence (considered the ‘true’ sequence) at the flow level, we can identify examples of over-calling a zero-length homopolymer (Flow Cycle #2), and under-calling a one-length homopolymer (Flow Cycle # 6). Flow Cycle #5 demonstrates a zero-length homopolymer being correctly called as zero.

It has been previously reported that ‘homopolymer errors’ (a term originating from Roche 454 pyrosequencing) are the dominant error type in PGM data [2], [3]. Homopolymer errors are a consequence of inaccurate flow-values resulting in over (insertion/s) or under-calling (deletion/s) the length of homopolymeric regions. For consistency with the literature, we refer to these as homopolymer errors, but emphasise that over and under-called flows are not always associated with homopolymers of length two or more [13], illustrated in **Figure 4**. This distinction is particularly pertinent for PGM data, where over-calling any flow-position can lead to the insertion of additional bases, even in flows where a ‘negative flow-value’ (<0.50) should have been called.

On average, 52.5% of reads generated with the OneTouch 100 bp kit had no observed homopolymer errors. Using the 200 bp manual kit, the average percentage of error-free reads drops to 8.3%, but this is markedly improved by using the 200 bp OneTouch kit, with ∼22.2% of reads, on average, free of homopolymer errors (**Figure S7**). Both 200 bp kits had a lower mean percentage of error-free reads when run on the 316 chip (4.4%–200 bp manual, 17.9%–200 bp OneTouch) versus the 314 (11.1%–200 bp manual, 26.7%–200 bp OneTouch). This difference suggests that even though only the size of the chip was varied, global well accuracy appears to be compromised by higher well densities. The 99^th^ percentile for the number of errors per read was between 11–14 for the 100 bp kit, 33–38 for the 200 bp manual kit and 32–38 for the 200 bp OneTouch kit.

**Figure 5:**
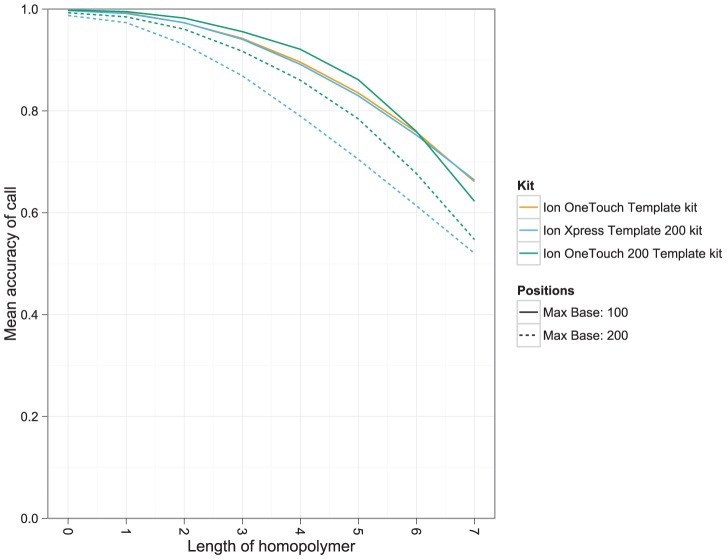
Calling accuracy decreases with homopolymer length. Lines show mean accuracy for each kit by reference homopolymer length, across bases 10–100 and bases 10–200, the latter range only relevant for the two 200 bp kits.

As with Roche 454 [11], [14], [15], base-calling accuracy decreases with the length of the homopolymer, with 6-mer calling accuracy approaching 68%±6.9% s.d using the OneTouch 200 bp kit (**Figure 5**). However, unlike Roche 454, insertions are more likely than deletions when the ‘homopolymer’ length is less than two (**Figure S8**). In fact, the accuracy of calling a zero length homopolymer was between 98.75% and 99.72% across the kits (a substantial contributor to the overall high insertion-rate). While insertions are the dominant error-type, they are more common for homopolymer lengths less than two, but are rapidly overtaken by deletions for homopolymers of length two or more (**Figure S8**). The accuracy in calling a homopolymer is highly variable, even for short homopolymers (s.d of 0.5%–1.7% for 2-mer accuracy) suggesting that base-calling accuracy is not dependent on homopolymer length alone.

While initially measured at the base level, over-call/under-call errors are the consequence of inaccuracy at the flow-value level. Thus, relationships between error occurrence and features of the read and sequencing parameters are best examined directly at the flow-value level [15]. Inspection of the error rate by flow position also shows a positive correlation between the homopolymer error rate and the flow position (**Figure S9**). However, the error rates per flow are substantially higher than those observed per base, with homopolymer error rates approaching 20–30% at flow position 350 onwards for the 200 bp One Touch kit. Surprisingly, there is also an observed periodicity in the error rate, with the period remaining approximately constant, however the amplitude increases with distance from the first flow. Intuitively, this periodicity suggests that the error rate may be influenced by the position-within-a-flow cycle (PIC) in a non-linear way. Inspection of the relationship between PIC and error rate suggested no obvious parameterised function, so each PIC (0–31) is considered a different ‘factor’.

Rather than directly modelling the errors, a double-generalised linear model (DGLM) was fitted to the flow-values. For a given homopolymer length and other factors, flow-values were approximately Gaussian distributed, however with a non-homogenous dispersion; that is, the mean and variance showed dependence on the explanatory variables, particularly homopolymer length (**Table 3** **, Table S1**). The mean effects for species, chip, homopolymer length, flow cycle and PIC were found to be important, however the machine used, and the read X and Y coordinates were not, and subsequently were removed from the model (**Methods**). The same set of factors were also found to significantly contribute to the dispersion. The model can be expressed as
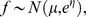
where 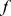 is the flow-value, 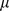 is the mean and 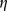 is the log-variance. Both the mean and log variance are linear-predictors, that is,
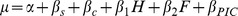
where here 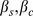 and 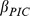 are coefficients taking a different value for each species, chip and PIC respectively; 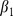 and 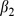 are multipliers of the homopolymer length and flow cycle respectively; and 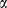 is a constant. 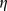 is similarly defined, with different values of the coefficients and the exponentiation to ensure a positive variance. Unlike Roche 454 pyrosequencing, where the prevalence of errors are a consequence of increasing variance in flow-calls (with insertions and deletions approximately equally likely [14]), it would seem that both the mean and variance are contributing to the over-call/under-call rate in Ion Torrent PGM sequences.

**Table 3:** Estimated main and deviance effects for each explanatory variable in the double-generalised linear model.

Variable	Coefficient (Mean effect)	P-value (Mean effect)	Coefficient (Dispersion)	P-Value (Dispersion)
Intercept	4.405×10^−02^	p<0.0001	−4.7120481	p<0.0001
Species (*S.tokodaii*)	−2.606×10^−03^	p<0.0001	0.0309340	p<0.0001
Chip 316	−2.527×10^−03^	p<0.0001	0.2013263	p<0.0001
200 bp Manual Kit	−3.864×10^−03^	p<0.0001	0.0815632	p<0.0001
200 bp OneTouch Kit	6.304×10^−03^	p<0.0001	−0.2425064	p<0.0001
Homopolymer Length	9.754×10^−01^	p<0.0001	0.4862209	p<0.0001
Cycle Number	−7.071×10^−04^	p<0.0001	0.1471443	p<0.0001

Position-in-cycle (PIC) effects are in **Table S1**. The intercept represents the mean effect (or dispersion effect) for an observation with all settings at baseline (baseline factors in this model taken to be *B. amyloliquefaciens*, 100 bp OneTouch Kit and Chip 314). The other coefficients are the differences from when their respective factor is changed from baseline.

As expected, the homopolymer length was the main contributor to the mean of the flow-value distribution (**Table 3**). Contrary to expectation, the coefficient for homopolymer length was not 1, but 0.975. Thus, with each additional base in a homopolymer, we see a decrease in the mean flow value of 0.025 (i.e. 0.975 main effect for a homopolymer of length 1, 1.95 for length 2 and so on). This shift downwards drives the increasing ‘gap’ between the deletion-rate versus the insertion-rate with increasing hompolymer length, consistent with our observation that deletions are the dominant error-type on longer homopolymers (**Figure S8**).

The cycle number imposed little influence on the mean of the flow-value distribution, but given its large dispersion coefficient and that it is a numeric variable as opposed to a factor, its contribution to the overall variance is the second largest from the third flow-cycle on. Interestingly, the chip, and kit made only small contributions to the mean of the flow-value distribution, but had large and substantial influences on the dispersion (**Table 3**). The newest template kit considered here, the 200 bp One Touch, exhibited significantly less dispersion (variance) in flow-value in contrast to the older kits.

**Figure 6:**
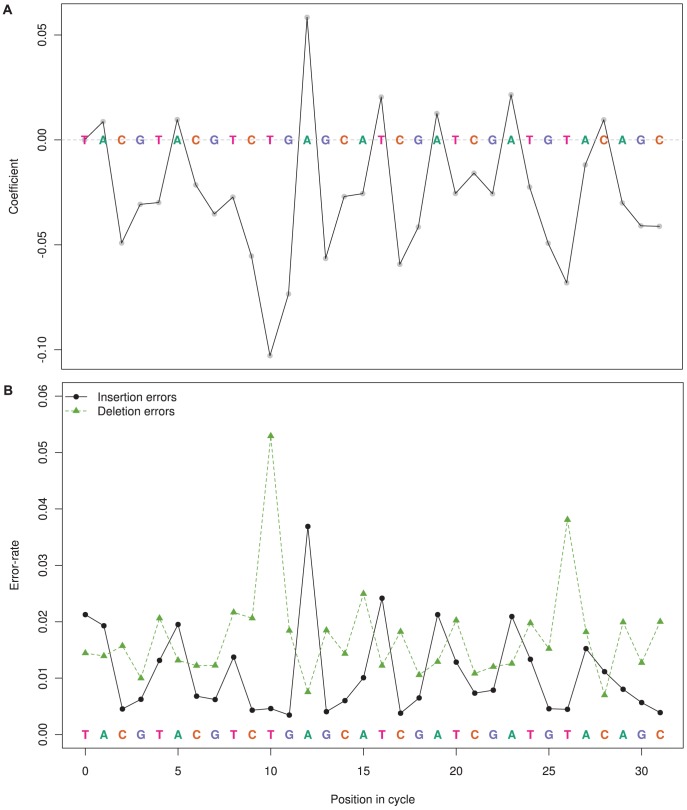
The influence of position in cycle (PIC, labelled 0–31) on flow-value distributions and consequently error rate and type. Panel (a) shows the coefficient (main-effect) of each flow cycle position as predictors of mean of the flow-value distribution. Panel (b) shows the error rate broken down by insertions and deletions for each PIC. These do not include flow-values for homopolymers where the reference homopolymer length is zero.

All PIC terms were found to significantly contribute to the mean, with some positions shifting the mean by −0.10 to +0.05, however the fitted effects were not consistent across PIC corresponding to the same nucleotide (**Figure 6a** **, Table S1**). This may explain why obvious groupings of nucleotides (GC versus AT, pyrmidines versus purines) as a factor in the DGLM were not significant. However, the three largest effects for a PIC are attributed to T or A flows, and the magnitude of these effects result in indel error rates up to double that of other PIC (**Figure 6b**). This suggests that sequencing of low G+C% species will result in a higher error rate than high G+C% species, consistent with the observation that *S. tokodaii* had a higher error rate than *B. amyloliquefaciens* (**Figure 3**). While PIC 10 and 12 had substantial mean-effects, the contribution of these factors to the dispersion was less than fitted for other PICS that had substantially smaller mean-effects. Thus, these PICs consistently introduce a shift to the mean flow-value by −0.10 and +0.05 for PICS 10 and 12 respectively.

A caveat of our modelling approach (using the Gaussian model) is that we could not include homopolymers of length zero due to the difficulty of modelling a normal which can only take on positive values. Based on the empirical error rates for over-called zero flows, we found this error rate (similar to longer homopolymers) increases by cycle, and in particular, is higher for PICS with a large positive coefficient in the model (i.e. PIC 12, followed by PICs 16, 23, 0 etc.), and very low for PICS with a negative coefficient (PIC 10, 13, 26 etc.) (**Figure S9**).

**Figure 7:**
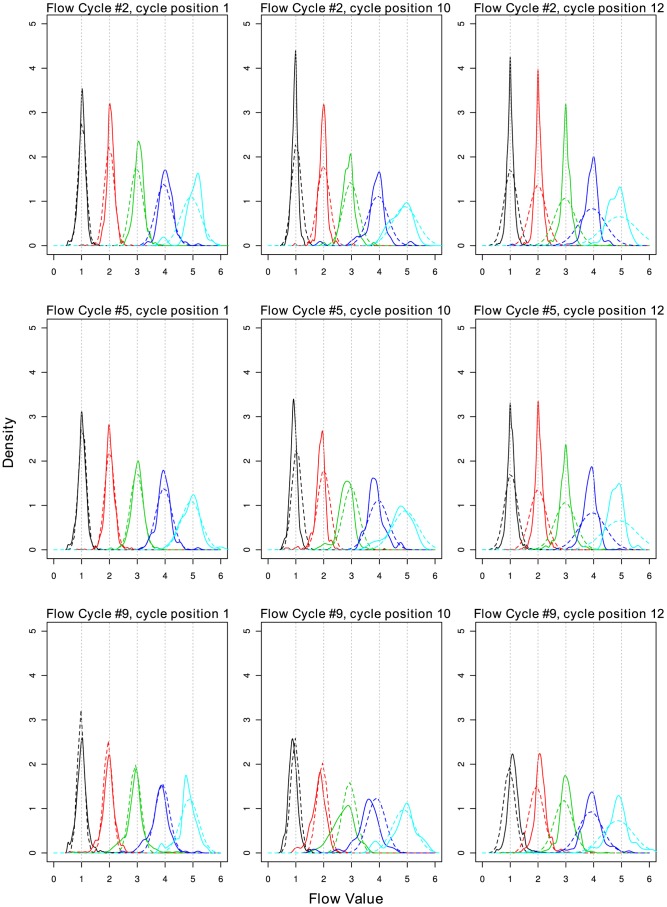
Comparison of predicted versus empirical distributions of flow-values for homopolymers of length 1–5. Predicted (solid line) and empirical distributions (dotted line) of flow-values for homopolymers of length 1–5 (colours - black, red, green, blue and teal), for flow cycles 2,5,9 (rows) and PIC 1,10 and 12 (columns) for species *B. amyloliquefaciens*. The low number of observations of homopolymers of length 5 is the likely cause for abnormal distributions for this homopolymer length. The ‘shoulders’ observed in the data are often due to unexpectedly high popularity of boundary flow-values (eg. 0.51, 1.49, 1.51…).

We evaluated the fitted model visually, comparing predicted density from the model versus the empirical distribution (**Figure 7**). Overall, we find the model fit is adequate, although we do note that there are some deviations from normality (less variance in earlier cycles, overpopular flow-values, assymetry). The empirical models are available from http://ecogenomic.org/acacia. As we have identified clear chip and kit effects, we recommend using the aforementioned models generated from the same combination of chip and kit as to be analysed.

### Substitution errors

The occurrence of substitution errors in Roche 454 pyrosequencing has previously been attributed to PCR processes [16], with an expected substitution error every 10^5^–10^6^ bases [17]. While PCR does contribute to the substitution rate, the substitution rates observed in this study (see above), can not be explained by this alone. We found that a disproportionately large number of substitution errors (between 16%–33% across the datasets) had a low-confidence flow-value call of 0.51. This is in contrast to the percentage of ‘correct’ 1-mer calls with a 0.51 flow-value call (0.4%). All substitutions with the flow-value 0.51 had a quality score lower than 20, 99% of them had a quality of 12 or less, thus quality scores can be a reasonable indicator of PGM introduced substitution errors.

**Figure 8:**
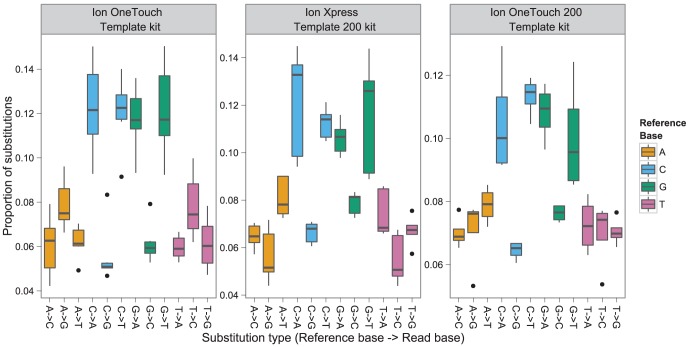
Breakdown of substitution type as a proportion of all substitutions for each sequencing kit.

While transitions (C <-> T, G <-> A) are the most prevalent form of substitution reported in bacteria [18], transitions from C to T and G to A were found to be equally likely with transversions from C to A and G to C for both species (**Figure 8**). As these rates have not been corrected for the G+C% of the parent organisms (both with <50% G+C), the proportion of substitutions which transition/transverse in higher G+C% organisms is likely larger than observed here. The novelty of this observation, in light of accepted substitution mechanisms, suggests that these substitutions are a consequence of the PGM sequencer, which is further reinforced by the prevalence of low confidence flow-calls for these substitutions. emPCR is an unlikely source of these errors, as transitions are the dominant substitution type for 454 GS FLX Titanium sequencing [16]. Considering that the PGM introduces substitution errors at a rate of between 0.04%–0.1%, rare variants (single nucleotide polymorphisms) which occur at a frequency greater than 0.1% may be detected using the PGM platform.

### A closer look at PGM quality scores

Each quality score, *q*, generated by the PGM base-caller is Phred-based, where *q* = −10×log_10_(*p*_error_). A quality score is assigned to each base using a pre-computed quality lookup-table distributed with each version of the PGM software. The lookup table uses six predictors of local quality, described elsewhere (Life Sciences Technical Note Version 2.0.1–2.20).

**Figure 9:**
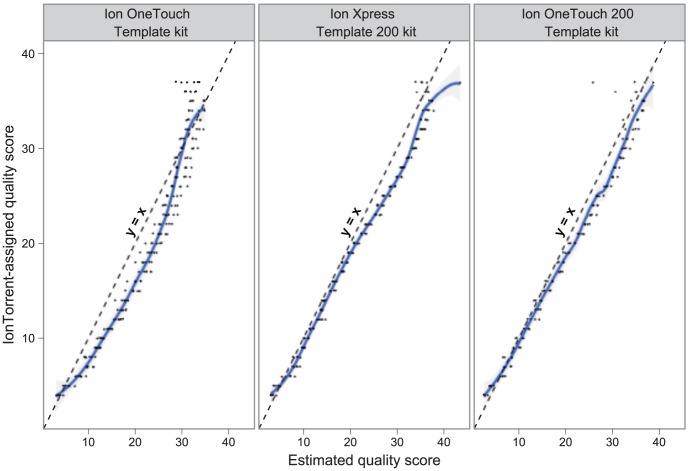
Ion Torrent quality scores versus empirically estimated quality score for base. The grey cloud surrounding the LOESS smoother function indicates the 95% confidence interval for the conditional mean. Individual observations for each quality are plotted as black points.

For each Ion Torrent quality score, we evaluated the empirical rate of error for bases assigned to that quality score. Consistent with previous reports [1], [2], we observe that the PGM quality scores underestimate the base accuracy, but observe that they have become more accurate with sequential sequencing kit releases (**Figure 9**). The relationship between the empirical quality and the estimated quality is not strictly linear, potentially a consequence of the quality score look-up table method. As with Roche 454 quality scores, the qualities can be only used to detect inserted and substituted bases [11]. We evaluated whether there was any relationship between position within the homopolymer and the assigned confidence. We found that even in correctly called bases within a homopolymer, there is a decrease in assigned quality along the homopolymer length (**Figure S10**), as was found in Roche 454 pyrosequencing [11], although, overall the quality scores decrease more rapidly for inserted (error) bases. Counter intuitively, it is possible for the first base in a homopolymer run to have *lower* quality score than later bases, irrespective of whether the homopolymer is the correct length or not (**Figure S10**). This could originate from penalties for local or environmental noise (P1 and P6 in the documentation), which allow individual base qualities to be adjusted if immediately upstream or downstream calls are noisy.

### How effective is Ion Torrent quality assurance?

The base-calling software in the Torrent Suite (version 2.0.1) performs two quality assurance steps prior to outputting sequences. The first step evaluates the residual between observed flow values and predicted flow values, based on a model of the flow cell. Reads with residuals that produce a median absolute value greater than a given threshold are filtered from both the SFF and the FASTQ, as they are assumed polyclonal. The second step scans non-polyclonal reads to identify undesirable 3′ regions of the read, which are subsequently trimmed. Undesirable regions are defined as regions containing the adapter sequence (and beyond) as well as low-quality regions. Should both adapter and quality trims be specified, the more stringent trimming is used. The newly released Torrent Suite (2.20) introduced a third trimming approach which clips the read based on high-residual ionogram (HRI) 1-mer and 2-mer flow values, which may indicate ‘noisy’ flows. A high-residual 1-mer is in the range [0.50, 0.59] or [1.40, 1.49], a high residual 2-mer in the range [1.50, 1.59] or [2.41, 2.49] (Torrent User Documentation Version 2.2.0).

We consider the influence of PGM quality and HRI trimming methods on error rates and other metrics for each of the sequencing kits (**Methods**, **Table 4**). For consistency, we initially apply our trimming, where we only consider the first 100 bp for the 100 bp OneTouch kit, and the first 200 bp for the 200 bp Manual and 200 bp OneTouch kits. We used the quality clip as specified in the SFF file, and calculated the clip points for HRI trimming. Very little improvement was gained by applying the quality clip after our analysis trimming. This suggests that only unmappable reads are removed by PGM quality clipping, and/or quality trimming is occurring after base 100 for the 100 bp OneTouch kit, and 200 for the 200 bp kits. This is to be expected, considering that only a long, consistently noisy region of the read could violate the quality threshold (mean of Q9 or less across 30 bp, i.e. 87.5% mean base accuracy). The addition of HRI trimming results in a substantial improvement over our ‘analysis’ treatment, with an absolute decrease in insertion and deletion rates by 0.23% to 1%, with generally greater improvements in the insertion rate over the deletion rate (**Table 4**). While there is asymmetry in the insertion versus deletion rate, we did not expect a disproportionate decrease in the insertion rate compared to the deletion rate after trimming. In an attempt to improve the deletion rate, we considered whether the addition of HRI 3-mers to this metric would account for our previous observation that deletions are more common in longer homopolymers, however this did not yield substantial improvements (**Table 4**).

**Table 4:** Effect of quality and flow trimming on dataset metrics, aggregated by kit used.

	Treatment	AT			AT+QT			AT+QT+HRI			AT+QT+HRI3		
	Kit	100	200M	200	100	200M	200	100	200M	200	100	200	200M
**Metric**													
Insertion Rate (%)		0.83	2.68	1.76	0.80	2.64	1.74	0.42	0.97	0.79	0.39	0.89	0.73
Deletion Rate		0.80	2.00	1.08	0.77	1.97	1.07	0.45	0.87	0.59	0.43	0.81	0.56
Comparison homopolymer rate					1.5% [2]	1.78 [3]							
Read length: 25th percentile		99	199	199	99	199	199	90.0	105.0	167.0	85.0	99.0	160.0
Read length: 50th percentile		990	199	199	99	199	199	98.0	170.0	198.0	98.0	164.0	198.0
Read length: 75th percentile		100	200.0	200	100.0	200	200	99	199	199	99	198.0	199.00
% of data retained		100.0	100.0	100.0	99.8	100.0	100.0	99.4	99.3	99.6	99.3	99.1	99.5
% error-free reads		52.5	8.3	22.2	53.3	8.4	22.2	63.4	24.1	29.6	65.0	27.0	31.0
Errors per read: 50th percentile		0	6	2	0	6	2	0	2	1	0	2	1
Errors per read: 75th percentile		2	13	7	2	13	7	1	4	3	1	3	3
Errors per read: 99th percentile		13	35	36	12	35	35	6	12	13	5	11	12

AT = Analysis trim, QT = Quality trim, HRI = High-residual ionogram trim (1-mers and 2-mers), HRI3 = High-residual ionogram trim (1-mer, 2-mer, 3-mers). The ‘comparison homopolymer rates’ are taken from other literature using the same kit and level of quality assurance (both cases used Torrent Server version 1.5.0).

After default HRI clipping, the vast majority of reads in the 200 bp kits contained at least one error (**Table 4**), with the top 99^th^ percentile as high as 6 for the 100 bp OneTouch kit, and 12–13 for both 200 bp kits (an error-rate of ∼6%). No obvious global metrics could be identified to filter these error-prone reads.

## Discussion

As with any new sequencing technology, exploring the nuances of the data lays an essential foundation for developing platform-specific bioinformatics methods. The goal of this study was to provide a comprehensive evaluation of the types of errors and biases introduced during PGM library preparation and sequencing. Previous studies which used the manufacturers aligner, ‘tmap’, on default settings implicitly removed reads with two or more indels prior to data analysis [2], [3]. As these filtered reads can only be distinguished with the benefit of a reference genome, such studies are more relevant for re-mapping applications. By using an indel-tolerant mapping approach, we were able to map almost 100% of the reads, which largely explains why the global base error-rates calculated in this study are higher than previously reported.

Even after applying the quality trimming used for the initial Ion Torrent studies, we found that our estimate of quality-clipped accuracy for the 100 bp OneTouch kit was substantially lower than first reported. The first Ion Torrent study was conducted prior to the changes in the flow-cycle pattern, a change implemented to improve synchronicity between reads on the same bead. By analysing the flow-values, we are able to show that this change in the flow-pattern results in specific positions within the flow cycle being more susceptible to over-call/under-call error than others. Error-prone cycles tend to be ‘A’ or ‘T’ flows, and unlike Roche 454, over-calls of zero length homopolymers and under-calling homopolymers of length one are not improbable. We find that insertions are more common than deletions overall, however, under-calling (deletions) rapidly becomes the dominant error type with increasing homopolymer length. The variance of flow-values with respect to homopolymer length, cycle number, and position-within a cycle (PIC) will present challenges to studies, such as indel variant detection, which often assume a global base error rate. Consequently, higher read coverage and use of PGM flow-specific error rates will be required for applications to confidently distinguish PGM indel errors from genuine variants. However, the substitution error rate, which occurs at an order of magnitude lower than the indel error rate (0.04%–0.1%), suggests that Ion Torrent could be used to detect SNP variants that occur at frequencies greater than 0.1%.

Through the use of replicates and modelling of flow-values, we are able to identify high frequency indel (HFI) errors that could easily be mistaken for polymorphisms in the absence of replicates. Flow-level analyses suggest that these errors do not correlate with factors implicated in over-call/under-call errors. The frequency of HFIs with respect to the reference genome will yield undesirable results for a number of applications. For example, in polymorphism detection, HFI regions will yield false positives [5]. In assembly, these regions yield unresolvable differences [2] or frame-shifts. Amplicon sequencing will suffer both from run-specific HFI errors and synchronised over-call/under-call errors; it is expected that amplicons from closely related species will be synchronised in their called flows, thus exposing them to much higher error rates as a result of noisy PICs. Given the difficulty of detecting HFIs in isolation, we recommend generating two or more datasets from the same starting DNA to resolve the majority of HFI errors (i.e. run-specific HFIs).

Consistent with previous results, we find the PGM introduces coverage bias against low and high G+C% sequences, with evidence suggesting that attempts to sequence high G+C% organisms (greater than 65% G+C%) may even fail during the PGM library preparation. Although the mechanisms behind these failed libraries are not evaluated here, we anticipate they will bias representation of high G+C% organisms in metagenomes and metatranscriptomes.

As expected for a new technology, there have been marked improvements in the PGM since its limited release in January, 2011. The newest kit considered in this study, the 200 bp OneTouch, has substantially reduced the error rates observed in the 200 bp Manual kit.

Furthermore, the accuracy of PGM quality scores has improved markedly with each kit release. The addition of HRI-based clipping (as of Torrent Server 2.2.0) to complement the relatively lax quality trim has proven extremely effective at removing the error-prone 3′ end of the reads, albeit at a cost of 20–30 bp of read length. For datasets processed with older versions of Torrent Server, we highly recommend re-running the PGM analysis to improve run quality. However, trimming cannot remove all errors. Thus, we recommend that researchers who intend to use or develop methods for analysing PGM data take into account that the PGM has a higher error-rate than both Illumina and 454, and that PGM global base error rates are poor substitutes for flow error rates. Armed with the models developed in this study, bioinformaticians can develop platform-specific approaches for the PGM that adequately account for the majority of errors introduced by this platform.

## Methods

### DNA

DNA for *Bacillus amyloliquefaciens* subsp. *amyloliquefaciens* DSM7, *Deinococcus maricopensis* DSM 21211 and *Sulfolobus tokodaii* DSM 16993, was acquired from the Leibniz-Institut DSMZ – German Collection of Microorganisms and Cell Cultures. These organisms were selected as they had small genomes consisting of a single chromosome, no plasmids, and complete reference genomes. They were also selected to span a range of mean genomic G+C% (*B. amyloliquefaciens* 46.1%, *D. maricopensis* 69.8%, *S. tokodaii* 32.8%). The SFF files for all *B. amyloliquefaciens* and *S. tokodaii* datasets are available from http://ecogenomic.org/acacia.

### Library preparation and sequencing

For the 100 bp libraries, 100 ng of genomic DNA was sheared by adaptive focused acoustics using a Covaris S2 (Covaris Inc.) and Covaris Micro-tubes and using the method and shearing conditions described in the Ion Fragment Library Kit (publication 4467320 Rev. B) protocol. For 200 bp libraries, 200 ng of genomic DNA was also sheared using a Covaris S2, with modification of the shearing conditions (intensity, 4; time, 27 sec). The remaining steps in the library preparation were performed using the Ion Plus Fragment Library Kit, using the corresponding User Guide (Publication 4471989 Rev. B), with modification. Due to the shearing volume required for the Covaris S2 micro-tubes, the sheared DNA samples were treated as if they were 1 µg samples, in the end repair steps, as this catered for a larger starting volume. From that point forward the library was treated as if the input was 100 ng. Following adapter ligation and nick repair, the library was size selected using a Pippin Prep (Sage Science) instrument, followed by 6 cycles of amplification.

Using the Agilent 2100 Bioanalyzer (Agilent Technologies) with the High Sensitivity DNA Kit (Agilent Technologies), the quality, size and concentration of the libraries was determined and the library was diluted, prior to template preparation, so as to keep polyclonal values in the sequencing results to a minimum. For 100 bp libraries, the Ion OneTouch system (composed of the Ion OneTouch Instrument and Ion OneTouch ES) was used to prepare the template, using the methods outlined in the Ion OneTouch Template Kit and associated User Guide (Publication 4468007 Rev. E). 200 bp libraries were prepared either manually, using the Ion Xpress Template 200 Kit and associated User Guide (Publication 4471974 Rev. C), or using the Ion OneTouch system, using the Ion OneTouch 200 Template Kit and associated User Guide (Publication 4472430 Rev C.).

100 bp templates were sequenced using the Ion Sequencing Kit and user guide (Publication 4469714 Rev. C). Manually prepared 200 bp templates were sequenced using the Ion Sequencing 200 Kit, using methods outlined in the corresponding User Guide (Publication 4471999 Rev. B), while 200 bp templates prepared using the OneTouch system were sequenced using the Ion PGM 200 Sequencing Kit and the associated User Guide (Publication 4474246 Rev. B). The Ion Torrent Suite 2.0.1 was used for all analyses and the SFF was subsequently downloaded for analysis. Test runs for *D. maricopensis* either failed to produce viable libraries, or produced very few reads of low quality, so sequencing this species was no longer pursued.

A factorial design was used for estimating variability due to Chip, Species and Kit, consisting initially of 8 sequencing runs (**Table S2**). A second experiment using a randomised Plackatt-Burman design consisting of 4 runs was conducted to help estimate inter-machine variability (**Table S3**). However, the Ion Xpress Template 200 kit was phased out during the experiment, preventing the completion of the final experiment. The release of a new kit part-way through the data-generation necessitated the addition of four new datasets, also conducted using a Plackatt-Burman design (**Table S4**). In total, 15 datasets were generated.

### Read preparation and alignment

The unclipped reads, flows and qualities were extracted from the SFF. The location of the predicted adapter sites for each read was also extracted. Only reads with an adapter site were clipped to the recommended length. All reads retained their flow key (the first four called bases in every read, TACG) to maintain ease of moving between a base and flow-value coordinate system. Flow and cycle coordinate systems reported start from zero, and base positions start from one.

The reference sequence for each genome was downloaded from NCBI, and converted to run-length encoded [19] (RLE) form (*S. tokodaii* - NC_003106.2, *B. amyloliquefaciens* NC_014551.1, and *D. maricopensis* NC_014958.1). Each read was also collapsed to its RLE form (i.e. homopolymers collapsed into a single base). This reduces the influence of numerous homopolymer errors on the alignment thresholds, as well as making it easier to transition between flow and base coordinates.

Each read was aligned to its respective reference genome using Segemehl version 0.1.2 [20], an InDel tolerant short-read aligner. The alignment output was parsed and the aligned regions extracted from both the read and reference RLEs. This was necessary as both substitutions and identical bases are reported as matches (‘M’) in the SAM format generated by this version of Segemehl. Using the SeqOp string and the aligned sequence segments, each position in the alignment was recorded as a match, substitution, insertion or deletion. Using a set of in-house Perl scripts and SQL databases, the alignment positions were mapped to their relevant flow-value, base position/s and quality scores. Read level attributes such as average quality, average G+C% in 100 bp windows and read length were also calculated. To avoid over-inflation of the error rate due to (1) 5′ misalignments and (2) homopolymers which overlapped the last base of the key and the first base/s of the read, we only consider base positions 10 and greater for analysis. Furthermore, analysis was restricted to the first 100 bp for the Ion OneTouch Template Kit (100 bp reads), and 200 bp for the Ion Xpress Template 200 and Ion OneTouch 200 Template kits, as the number of reads longer than this decreased rapidly leading to exaggerated error rates at these base positions.

### Statistical filtering and analysis

#### Repeats

MUMmer 3.22 [21] (program ‘repeat-match’ with parameter –n 30) was used to identify regions of perfect match repeats within the reference genomes. These regions were masked from all downstream analysis.

#### Polymorphisms

While the sequenced type strains for each species were used, there is still potential for polymorphic differences between the cultured strains and the reference genome. Classification and consequent masking of these genomic locations in the reference allows more accurate modeling of error rates in downstream analyses.

Beginning with substitution differences between the read and reference, we simply modeled the observed number of differences, *X*, at each reference position as a binomial, with the probability of substitution estimated from the data (mean substitution rate). Due to the large number of comparisons, the p-values were corrected using Holm's method [22]. The significance threshold was taken to be 0.05. A similar approach was adopted for detecting insertion and deletion polymorphisms, however, in the case of single-base insertions (over-calls of zero) the coverage of the site was taken to be the maximum coverage of the bases immediately adjacent to the insert. The probability of an insertion or deletion was also estimated from the data, but parameterised on the homopolymer length, as previous reports have shown that the error rate increases dramatically with homopolymer length [2].

Strand-asymmetry of high frequency indels was only evaluated on the indel polymorphisms found in the 200 bp kits (both Manual and OneTouch), and only for sites that had reads mapping in both orientations. For each site, the data form a 2×2 table, namely strand by presence/absence of indel. We therefore used Fisher's exact test to generate a p-value for every site, testing the null hypothesis that indel frequency at that site was not orientation specific. The p-values were then adjusted to control the family-wise error rate using Holm's method [22]. Only sites with corrected p-values smaller than 5% were considered to demonstrate significant evidence of orientation specific indel frequencies at that site. Using this approach, the orientation specificity may differ by site. (The analysis was repeated using a chi-squared test in place of Fisher's exact test with similar results).

#### Replicate bias

To prevent reference end-effects and repeat-effects, we used repeat-masked data and ignored the first and last 120 bp of the reference genome. We visually identified whether the distribution of read starting position (5′ aligned position) on the reference genome was uniformly distributed for each individual run.

#### G+C% bias in read coverage

To evaluate whether there was a relationship between G+C content and read depth, we calculated the average coverage of bases within disjoint 100 bp windows across the genome, as well as G+C% also calculated for these windows. Areas expected to have high or low coverage for processing reasons were masked from the analysis, these included the first and last 120b of the reference genome, as well as genomic 100 bp bins that contained repetitive sequences. The coverage was normalised for each run by dividing the coverage in each window by the mean coverage across all windows for that run. A square-root transformation was applied to the run-normalised coverage. After initial inspection, we identified that a number of very large coverage values were the result of an un-masked LSU rRNA in the *B. amyloliquefaciens* genome. This small region was masked prior to G+C modeling. The relationship between the square-root normalised coverage and G+C% content was evaluated by fitting linear models using the *lm* function in the R statistical package.

#### Error rates

Base-error rates were calculated as the number of errors in the alignment, divided by the length of the alignment. This was to ensure that deletion errors, which are quite common on this platform, would be reflected by the error rate. Flow-error rates were calculated as the number of incorrect flow-calls divided by the total number of flow-calls. For specific break-downs (such as error rate for homopolymer of length X), it was the number of miscalled X-mer flows, divided by the total number of flows which were, or should have been, an X-mer, according to the reference genome.

#### Modeling flow values

Given RAM restrictions, a random subset of 18 million observations (flows) were sampled from all datasets as input to model fitting. Note that true zero calls and over-calls of a zero were not included in the model, as zero-flows were unlikely to be well-approximated by a Gaussian. The flow-values were then modeled as normally distributed, using a variety of read attributes (including chip, kit, machine, flow position, well x-coordinate, well y-coordinate, nucleotide, position in cycle (PIC), nucleotide, pyrimidine versus purine). As the flow-values for each homopolymer length did not share a constant variance, these needed to be modeled using a double generalised linear model (DGLM), which simultaneously models the mean and dispersion. In the DGLM used here, the mean was a Gaussian linear model and the dispersion was linear on a log-scale. Only terms with an effect size greater than 0.001 were retained in the model. While the PIC showed the strongest relationship with the flow error-rate, we considered the replacement of PIC with simpler terms, such as the nucleotide flowed or pyrimidine versus purine, however this was detrimental to the model. Based on these choices, a simpler model was created and assessed against the full-model for significance using ANOVA. Some terms removed were statistically significant (x-pos, y-pos, machine), however were practically unimportant contributing only a very small amount to the modeled flow value. We emphasise that the purpose of this statistical model is not to test for significant factors, but to produce usable predictions.

#### Quality score analysis

Each run was analysed individually to identify the accuracy of empirical quality scores. For each Ion Torrent quality score, we examined the error rate of bases assigned that score, and calculated the associated Phred score based on the error rate. To analyse the relationship between quality score and position within homopolymer, we sampled 20,000 reads from each run, and calculated the relative change in quality score from the first base to later bases within each homopolymer. We also recorded whether each of these consecutive bases was a correct call or an over-call.

#### Quality trimming

The PGM quality clip was extracted from the SFF file produced from Torrent Server 2.0.1. To emulate the PGM HRI trim approach, we calculated the percentage of HRI 1-mers and 2-mers out of the total 1-mer and 2-mer calls in the read, and continued clipping from the 3′ end until this percentage reached 3% or less (Torrent User Documentation 2.20). Our third HRI trim approach included HRI 3-mers (defined as flow-values in [2.50, 2.59] or [3.40, 3.49] in this calculation. As with the Torrent Server software, reads shorter than 4 bp after clipping were ignored.

#### Graphs

Graphics used throughout this manuscript were produced either using the R base package or ggplot2 package [23].

## Supporting Information

Figure S1 **Read length density functions for kit and species.** Note that both One Touch kits produce a small peak around 100 bp longer than the mode length.
(EPS)

Figure S2 **Intersection of HFI sites across 200 bp runs for each species.** Panel **a**) shows the overlap between HFI sites detected in *B. amyloliquefaciens* 200 bp runs, and panel **b**) shows the HFI site overlaps for *S. tokodaii* 200 bp runs, panel, **c**) shows the overlap between strand-asymmetric HFI sites detected in *B. amyloliquefaciens* 200 bp runs, and **d**) shows the overlap between strand-asymmetric HFI sites detected in *S. tokodaii* 200 bp runs. Red font highlights the HFI instances unique to a given run.
(EPS)

Figure S3 **Type and frequency of HFI by indel type.** Panel **a**) shows the HFI instances across all 200 bp runs (both species), broken down by error type and nucleotide, and **b**) shows the strand-asymmetric HFI instances broken down by error type and nucleotide.
(EPS)

Figure S4 **Linear model fit diagnostics plots for G+C% versus coverage.** Using a subset of data-points, these plots show the standard linear model diagnostics for the G+C versus coverage linear model. The data is not strictly normal as the response variable (coverage) is based on count data. The small number of zero coverage regions are the outliers in the ‘Residual versus Fitted’ plot, and the deviation from the normal quantiles in the ‘Normal Q-Q’ plot. Unmasked repetitive regions are the likely cause for outliers with high leverage ‘Residuals versus Leverage’ plot.
(TIFF)

Figure S5 **Smoothed normalised coverage versus proportion G+C content in 100 bp windows for three species.**
(EPS)

Figure S6 **Number of errors found within reads.** These plots exclude reads with more than 30 errors.
(EPS)

Figure S7 **Error-rate for insertions versus deletions by homopolymer length.** Rows correspond to restriction on base position considered for error-rate (maximum base 100 versus maximum base 200) Columns correspond to the kit analysed. Observations are the error-rate by flow position for the respective homopolymer length.
(EPS)

Figure S8 **Relationship between flow-call error-rate and flow position.** It is clear from these figures that the main effect is increasing with the number of flow cycles. The 316 chip has a higher error rate than the 314, and *S. tokodaii* is more error-prone than *B. amyloliquefaciens*. Periodicity is observed in the error rate with a peak and trough occurring regularly in each plot.
(EPS)

Figure S9 **Over-call rates for zero length homopolymer by position-in-cycle (PIC).** Consistent with our DGLM based on calls for 1-mers or longer, we find that over-calls of zero occur in the PICs with a positive coefficient in the model. Most notable is position-in-cycle 12.
(EPS)

Figure S10 **Relationship between quality scores and position in homopolymer.** The x-axis shows the base number in the homopolymer, the y-axis shows the relative change (qual(base 1) – qual(base x)/qual(base 1)) from the quality of the first base.
(EPS)

Table S1 **Coefficients for main and dispersion for position-in-cycle (PIC) effects in generalised linear model for flow-values.**
(DOCX)

Table S2 **Sequencing runs in the full-factor design.**
(DOCX)

Table S3 **Randomised Plackatt-Burman design for sequencing runs using OneTouch 100 kit and Ion Express Template kit.**
(DOCX)

Table S4 **Randomised Plackatt-Burman design for sequencing runs using OneTouch 200 bp kit.**
(DOCX)
