# Supplementary figures and images for: Shining a Light on Dark Sequencing: Characterising Errors in Ion Torrent PGM Data
Source: PLoS Comput Biol. 2013 Apr 11;9(4):e1003031. doi: 10.1371/journal.pcbi.1003031 (PMC3623719; doi:10.1371/journal.pcbi.1003031)

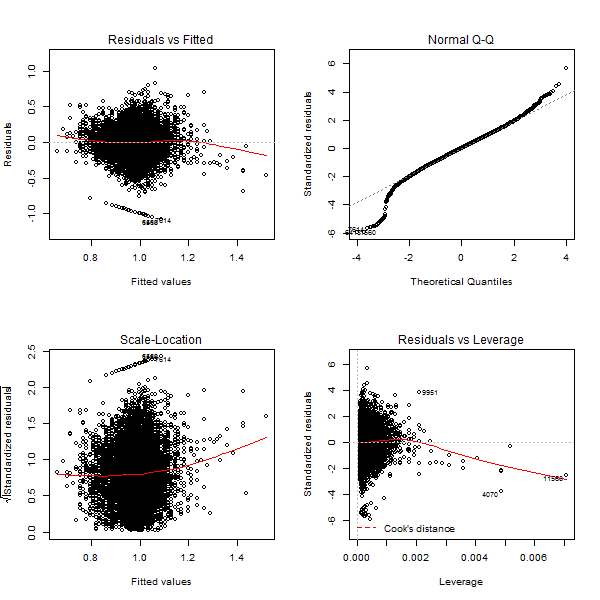

Supplement: Figure S4 — Linear model fit diagnostics plots for G+C% versus coverage. Using a subset of data-points, these plots show the standard linear model diagnostics for the G+C versus coverage linear model. The data is not strictly normal as the response variable (coverage) is based on count data. The small number of zero coverage regions are the outliers in the ‘Residual versus Fitted’ plot, and the deviation from the normal quantiles in the ‘Normal Q-Q’ plot. Unmasked repetitive regions are the likely cause for outliers with high leverage ‘Residuals versus Leverage’ plot. (TIFF) [file pcbi.1003031.s004.tiff]
