# Supplementary material for: Shining a Light on Dark Sequencing: Characterising Errors in Ion Torrent PGM Data
Source: PLoS Comput Biol. 2013 Apr 11;9(4):e1003031. doi: 10.1371/journal.pcbi.1003031 (PMC3623719; doi:10.1371/journal.pcbi.1003031)
Supplement: Table S1 — Coefficients for main and dispersion for position-in-cycle (PIC) effects in generalised linear model for flow-values. (DOCX) [file pcbi.1003031.s011.docx]

| **Variable** | **Coefficient**  **(Main effect)** | **P-value**  **(Main effect)** | **Coefficient**  **(Dispersion)** | **P-Value**  **(Dispersion)** |
| --- | --- | --- | --- | --- |
| Pos In Cycle 1 | 8.712e-03 | p < 0.0001 | 0.0057742 | 0.612069 |
| Pos In Cycle 2 | -4.915e-02 | p < 0.0001 | -0.2077587 | p < 0.0001 |
| Pos In Cycle 3 | -3.073e-02 | p < 0.0001 | -0.2041580 | p < 0.0001 |
| Pos In Cycle 4 | -2.986e-02 | p < 0.0001 | -0.0298921 | 0.007929 |
| Pos In Cycle 5 | 9.690e-03 | p < 0.0001 | 0.0182879 | 0. 101583 |
| Pos In Cycle 6 | -2.147e-02 | p < 0.0001 | -0.1278759 | p < 0.0001 |
| Pos In Cycle 7 | -3.529e-02 | p < 0.0001 | -0.1289092 | p < 0.0001 |
| Pos In Cycle 8 | -2.725e-02 | p < 0.0001 | 0.0043478 | 0. 698032 |
| Pos In Cycle 9 | -5.532e-02 | p < 0.0001 | -0.0894774 | p < 0.0001 |
| Pos In Cycle 10 | -1.029e-01 | p < 0.0001 | 0.0418692 | 0. 037314 |
| Pos In Cycle 11 | -7.343e-02 | p < 0.0001 | -0.1772494 | p < 0.000 |
| Pos In Cycle 12 | 5.843e-02 | p < 0.0001 | 0.1331877 | p < 0.0001 |
| Pos In Cycle 13 | -5.653e-02 | p < 0.0001 | -0.1294153 | p < 0.0001 |
| Pos In Cycle 14 | -2.698e-02 | p < 0.0001 | -0.1095077 | p < 0.0001 |
| Pos In Cycle 15 | -2.561e-02 | p < 0.0001 | 0.0403077 | 0. 007392 |
| Pos In Cycle 16 | 2.044e-02 | p < 0.0001 | 0.0361334 | 0. 000431 |
| Pos In Cycle 17 | -5.933e-02 | p < 0.0001 | -0.1434537 | p < 0.0001 |
| Pos In Cycle 18 | -4.152e-02 | p < 0.0001 | -0.1292816 | p < 0.0001 |
| Pos In Cycle 19 | 1.261e-02 | p < 0.0001 | 0.1394426 | p < 0.0001 |
