# Supplementary material for: Shining a Light on Dark Sequencing: Characterising Errors in Ion Torrent PGM Data
Source: PLoS Comput Biol. 2013 Apr 11;9(4):e1003031. doi: 10.1371/journal.pcbi.1003031 (PMC3623719; doi:10.1371/journal.pcbi.1003031)
Supplement: Table S2 — Sequencing runs in the full-factor design. (DOCX) [file pcbi.1003031.s012.docx]

| Id | Chip | Organism | Machine | Sequence Kit |
| --- | --- | --- | --- | --- |
| 314-B-a-100 | 314 | *B.*  *amyloliquefaciens* | a | Ion OneTouch Template Kit |
| 314-S-a-100 | 314 | *S.*  *tokodaii* | a | Ion OneTouch Template Kit |
| 314-B-a-200M | 314 | *B.*  *amyloliquefaciens* | a | Ion Xpress Template 200 kit |
| 314-S-a-200M | 314 | *S.*  *tokodaii* | a | Ion Xpress Template 200 kit |
| 316-B-a-100 | 316 | *B.*  *amyloliquefaciens* | a | Ion OneTouch Template Kit |
| 316-S-a-100 | 316 | *S.*  *tokodaii* | a | Ion OneTouch Template Kit |
| 316-B-a-200M | 316 | *B.*  *amyloliquefaciens* | a | Ion Xpress Template 200 kit |
| 316-S-a-200M | 316 | *S.*  *tokodaii* | a | Ion Xpress Template 200 kit |
