# Supplementary material for: Shining a Light on Dark Sequencing: Characterising Errors in Ion Torrent PGM Data
Source: PLoS Comput Biol. 2013 Apr 11;9(4):e1003031. doi: 10.1371/journal.pcbi.1003031 (PMC3623719; doi:10.1371/journal.pcbi.1003031)
Supplement: Table S3 — Randomised Plackatt-Burman design for sequencing runs using OneTouch 100 kit and Ion Express Template kit. (DOCX) [file pcbi.1003031.s013.docx]

| Name/s | Chip | Organism | Machine | Library Kit |
| --- | --- | --- | --- | --- |
| 316-S-b-100 | 316 | *S.*  *tokodaii* | b | Ion OneTouch Template Kit |
| 314-S-b-200M | 314 | *S.*  *tokodaii* | b | Ion Xpress Template 200 kit |
| 314-B-b-100 | 314 | *B.*  *amyloliquefaciens* | b | Ion OneTouch Template Kit |
|  |  |  |  |  |
